# Inclusive and sustainable active mobility: recommendations for older adults in Montenegro

**DOI:** 10.3389/fphys.2025.1752663

**Published:** 2026-01-12

**Authors:** Stevo Popovic, Gonul Babayigit Irez, Bojan Masanovic

**Affiliations:** 1 University of Montenegro, Faculty for sport and Physical Education, Niksic, Montenegro; 2 Western Balkan sport Innovation Lab, Podgorica, Montenegro; 3 Korea University, Seoul, Republic of Korea; 4 Mugla University, Muğla, Türkiye

**Keywords:** accessibility, active mobility, age-friendly cities, Montenegro, older adults, public health, sustainable urban development

## Abstract

This paper provides a perspective on inclusive and sustainable active mobility solutions for older adults in Montenegro, informed by research conducted in Podgorica and the wider region. Active mobility is a key factor influencing healthy ageing, social inclusion, and quality of life; however, its improvement requires a systemic approach connecting urban planning, public health, and social policy. In Montenegro, rapid urbanization, insufficient infrastructure, and limited intersectoral cooperation challenge the development of safe and accessible mobility options for older citizens. Using an analytical framework developed within two COST Actions and guided by the WHO “Age-friendly Cities” model, this perspective proposes recommendations aimed at improving accessibility, safety, and participation of older adults in public space. Special emphasis is placed on integrating GIS tools into local planning, introducing regular accessibility assessments, and involving older residents in decision-making processes. This perspective argues that inclusive and sustainable active mobility is not only an infrastructural requirement but also a public health priority that demands long-term, coordinated strategies aligned with European standards. It thereby contributes to advancing policy and planning efforts toward age-friendly urban development in emerging European contexts.

## Introduction

Active mobility, primarily walking and cycling but also other forms of everyday movement, is recognized as a key component of healthy ageing and sustainable urban systems (Götschi et al., 2022). Its importance extends beyond transport, contributing to physical and mental health as well as social inclusion. Promoting active mobility also reduces carbon emissions and enhances urban quality of life. European public health and urban development frameworks, such as the WHO Global Age-friendly Cities model and the EU Urban Agenda, place active mobility at the center of integrated policies connecting transport, health, and demographic change, which highlights the need for adapting these standards to Montenegro.

Montenegro faces long-term challenges limiting the development of inclusive mobility solutions for older adults. Demographic projections show rapid population ageing (Statistical Office of Montenegro, 2024), while the spatial and infrastructural design of most cities remains insufficiently adapted to the needs of older people. Incomplete sidewalk networks, limited safe crossings, few public benches, and poor lighting reduce their ability to move independently and safely. In addition to physical barriers, institutional obstacles persist due to weak coordination between transport, health, social care, and urban planning sectors, hindering comprehensive and sustainable solutions.

This paper builds on research conducted in Podgorica within the BOPALiM COST Action (CA23101), aimed at identifying inclusive and sustainable active mobility approaches for older adults in the capital. Key outputs (Brown and Kytta, 2014), especially GIS-based assessments, participatory mapping, and stakeholder engagement, provide valuable knowledge applicable to the national context. Comparing regional experiences also helps recognize shared challenges and identify good practices to support active and healthy ageing policies in Montenegro and neighboring countries.

The aim of this paper is to offer a public health and sustainable urban development perspective, together with recommendations for improving active mobility among older adults in Montenegro. The focus is on solutions that integrate physical accessibility, social inclusion, and institutional sustainability while aligning local policies with European and international standards. This perspective also seeks to support scientific and professional discussions on integrated mobility and ageing, and encourage practical, scalable models suitable for Montenegrin cities. Rather than conducting an original empirical analysis, this paper adopts a perspective-based and conceptual approach. It synthesizes existing European practices, policy frameworks, and prior research to propose strategic recommendations for improving inclusive and sustainable active mobility among older adults in Montenegro. The proposed framework is intended to inform policy development and guide future empirical research and pilot interventions, rather than to evaluate implemented measures.

## Current context and challenges in Montenegro

Although strategic planning has improved and the need for sustainable mobility is recognized, Montenegro still faces major obstacles that restrict active movement among older adults. Urban areas in most cities, such as Podgorica, Niksic, and Bar, have been shaped to prioritize motorized transport (Vujadinovic et al., 2021), while pedestrian needs, especially of older citizens, remain marginalized. This results in uneven sidewalk availability, discontinuous pedestrian routes, and breaks in public space (Masoumi et al., 2025).

Infrastructure barriers are a primary concern: sidewalks are often narrow, damaged, or blocked by vehicles, and pedestrian crossings are rarely adapted for reduced mobility. Inadequate enforcement of regulations allows these problems to persist. The lack of benches and resting places further limits participation in daily activities, reducing physical activity, independence, and social interaction. These issues are especially prominent in older town centers and suburban settlements where spatial design is far from modern accessibility principles (Rovcanin Premovic, 2024).

Institutionally, despite documents such as the National Strategy on Older Adults (Government of Montenegro, 2017) and the Sustainable Urban Development Strategy (Government ofMontenegro, 2022), implementation of mobility measures remains weak. Limited cooperation among health, transport, social care, and urban planning sectors leads to fragmented interventions, isolated ramps, partial sidewalk repairs, or seasonal campaigns, without a long-term, systemic approach to improving safety and accessibility.

Socio-cultural factors further complicate progress. Mobility of older adults is rarely treated as a public health priority, except in academic contributions from national institutions (Markovic et al., 2025). Older citizens are often perceived as passive users rather than active contributors to community design, reducing their influence and participation. Low trust and underdeveloped participation mechanisms hinder the adoption of inclusive policies that modern society increasingly requires.

Economic and demographic constraints also play a role. Smaller municipalities often lack financial and technical capacity to implement integrated mobility projects (Government ofMontenegro, 2018). Meanwhile, rapid ageing increases the need for accessible and socially connected environments. Without strategic and coordinated action, social isolation and reduced quality of life will deepen, raising both physical and mental health risks and the associated costs of care.

Montenegro is therefore at a turning point, between acknowledging the importance of active mobility and implementing it in practice. Introducing systematic accessibility assessments, expanding the use of modern tools such as GIS mapping, and strengthening the participation of older citizens are essential steps toward more sustainable, healthier, and equitable urban development.

## Experiences from the region and beyond and their potential application in Montenegro

Examples from Europe and the region show that active mobility for older adults can be successfully improved through combined infrastructural, educational, and institutional measures. Cities such as Ljubljana, Zagreb, and Sarajevo, as well as Vienna, Helsinki, and Copenhagen, demonstrate that systematic approaches based on cross-sector cooperation and citizen engagement can significantly enhance accessibility and urban quality of life.

In Slovenia, Ljubljana has integrated age-friendly principles into key areas of urban planning through its participation in the WHO Global Network for Age-friendly Cities and Communities and long-term action plans (Slana et al., 2013; WHO, n.d.-a). Improved pedestrian paths, adapted crossings, and multifunctional public spaces encourage movement and social interaction. Success has been driven by ongoing collaboration between local authorities, healthcare institutions, and civil society, ensuring decisions reflect community needs.

Vienna’s “Age-friendly City” model relies on detailed spatial assessments, regular surveys among older residents, and participatory workshops (WHO, n.d.-b). This approach identifies specific locations requiring improvements, such as additional benches, better lighting, or safer crossings. Alongside infrastructure, Vienna supports social mobility programs, guided walks and group activities, which foster physical activity and social connections.

Helsinki and Copenhagen highlight the close link between active mobility and public health policy (Saidla, 2018). Their strategies recognize mobility as a factor that prevents chronic diseases and improves mental health and overall quality of life.

In the Western Balkans, initiatives in Zagreb and Sarajevo combine spatial improvements with education and volunteer support for older citizens (Masoumi et al., 2025). Though still developing, they show growing regional commitment to integrating active ageing into urban policy.

These experiences offer valuable guidance for Montenegro. Key success factors include horizontal policy integration, connecting health, transport, urban planning, and social protection, along with mechanisms that enable older adults to actively participate in evaluating and shaping public spaces. Examples from Vienna and Ljubljana confirm that even small, well-planned interventions can significantly improve public health and daily living.

Adapting these principles to Montenegro requires adjustment to local conditions, especially limited resources in smaller municipalities. However, with support from regional experience and collaboration among communities, universities, and civil organizations, Montenegro can develop its own inclusive and sustainable model of active mobility aligned with European standards for healthy ageing.

## Recommendations for Montenegro

**FIGURE 1:**
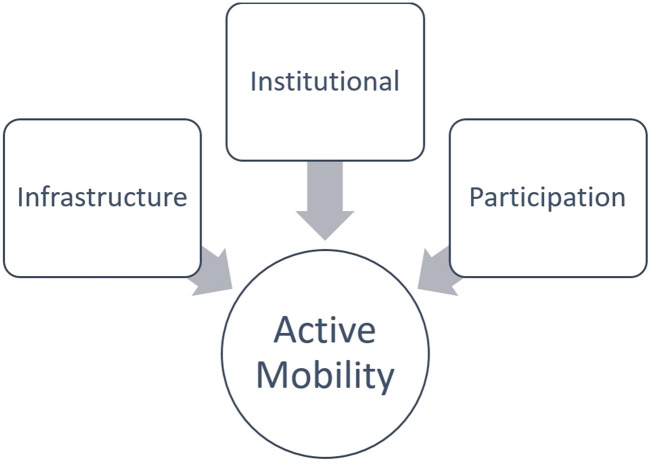
Core pillars influencing inclusive and sustainable active mobility of older adults.

Developing inclusive and sustainable active mobility in Montenegro requires a comprehensive approach that addresses infrastructural, institutional, and social dimensions. Based on European and regional experiences and analysis of the national context, three strategic directions are identified (Figure 1) as being of key importance:

- Improving physical accessibility,
- Strengthening institutions and policy coordination, and
- Enhancing participation and social inclusion of older adults.

These directions represent recommendations derived from comparative analysis of European practices and conceptual public health frameworks and do not constitute the results of an empirical evaluation conducted within this study.

Improving accessibility begins with creating a safe and comfortable pedestrian environment, especially for older people. Key measures include systematic assessment and mapping of sidewalks, crossings, and public spaces to identify barriers; integrating minimum accessibility standards (e.g., width, slope, lighting, visual markings) into local plans; installing benches and resting areas every 100–150 m in busy zones; and improving lighting and safety in pedestrian areas, particularly at night. Age-friendly micro mobility options, such as controlled-speed e-scooters and stable public bicycles, should also be considered. Wider use of GIS tools would help municipalities identify priority interventions and monitor progress with transparent, data-driven planning.

Institutional improvements require better coordination between transport, health, spatial planning, and social protection sectors. Establishing national and local intersectoral working groups could ensure integration of active mobility into public health and sustainable development policies. Further actions include developing indicators to monitor accessibility, training local planners in age-friendly design, and strengthening cooperation with universities and research centers that can support pilot projects. Harmonizing policies with European frameworks would also improve access to technical and financial support.

Enhancing participation is crucial for sustainable outcomes. Older adults should have an active role in planning public spaces rather than being viewed only as service users. Recommended measures include creating advisory councils of older adults at municipal level, organizing workshops, focus groups, and “walkability audits,” as well as launching public campaigns promoting active ageing. Intergenerational and volunteer initiatives can further support older citizens in overcoming mobility challenges, reinforcing community cohesion and shared responsibility for public space.

Successful implementation requires political commitment, technical capacity, and long-term dedication. Yet, evidence from European cities shows that even small, low-cost measures can yield strong benefits when strategically applied. For Montenegro, advancing active mobility among older adults represents an important step toward a healthier and more inclusive society aligned with European principles of sustainability and equal opportunities.

## Discussion and future outlook

Active mobility of older adults is a key yet often overlooked aspect of public health and sustainable urban development (Patil et al., 2022). As highlighted throughout this paper, it goes beyond infrastructure and serves as an indicator of quality of life, social inclusion, and intergenerational solidarity. European and regional experiences show that the most successful models rely on integrated approaches linking spatial planning, health, social policy, and active citizenship (Bechtold et al., 2021). Montenegro has the opportunity to shape its own model of active mobility, adapted to local conditions yet aligned with European standards.

The framework presented in this paper should therefore be understood as a strategic and conceptual contribution. While it does not empirically test specific interventions, it identifies priority domains and mechanisms through which inclusive active mobility policies may be developed and subsequently evaluated.

From a public health perspective, improving active mobility reduces chronic disease risk, strengthens functional independence and mental health, and lowers healthcare costs (Maresova et al., 2023). Encouraging walking, outdoor activity, and social interaction also reduces feelings of isolation and loneliness (Ihara et al., 2022), major challenges of population ageing. Thus, active mobility becomes a bridge between prevention and social inclusion.

With regard to sustainability, prioritizing pedestrians and cyclists can reduce emissions, ease traffic congestion, and support healthier urban environments. In Montenegro’s small and medium-sized municipalities, short distances make these benefits achievable with relatively low investments. Long-term results, however, depend on institutional cooperation and community engagement in planning processes.

Future steps should include establishing a national accessibility assessment framework to monitor progress and compare municipalities. Local pilot projects, such as “green corridors” for older adults, improved shading and seating, or guided community walks, can demonstrate tangible benefits and inspire broader implementation.

Regionally, Montenegro could take a proactive role in developing an age-friendly cities network within the Western Balkans, strengthening cooperation and data exchange. Participation in existing international networks would provide valuable institutional and technical support for long-term initiatives.

Future research should empirically assess the proposed recommendations through pilot projects in selected Montenegrin municipalities. Potential approaches include GIS-based accessibility mapping, participatory walkability audits involving older adults, and longitudinal studies examining changes in physical activity levels, social participation, and health outcomes. Such empirical evidence would be essential for validating, refining, and scaling inclusive and sustainable active mobility interventions.

This study is limited by its perspective-based design and the absence of original empirical data. Consequently, the proposed recommendations have not been empirically validated within the scope of this article. However, the paper provides a structured conceptual framework that can inform and support the design of future empirical studies and pilot interventions.

Ultimately, inclusive and sustainable active mobility should not be viewed as a secondary urban issue, but as a foundation of modern public health and social policy. Through strategic planning, intersectoral collaboration, and citizen participation, Montenegro can become an example of a society that supports healthy ageing and equal access to public spaces for all generations.

## Data Availability

The original contributions presented in the study are included in the article/supplementary material, further inquiries can be directed to the corresponding author.
